# Cardiovascular magnetic resonance parameters of atherosclerotic plaque burden improve discrimination of prior major adverse cardiovascular events

**DOI:** 10.1186/1532-429X-11-10

**Published:** 2009-04-24

**Authors:** Venkatesh Mani, Paul Muntner, Samuel S Gidding, Silvia H Aguiar, Hamza El Aidi, Karen B Weinshelbaum, Hiroaki Taniguchi, Rob van der Geest, Johan HC Reiber, Sameer Bansilal, Michael Farkouh, Valentin Fuster, John E Postley, Mark Woodward, Zahi A Fayad

**Affiliations:** 1 Imaging Science Laboratories; Translational and Molecular Imaging Institute, Department of Radiology, Mount Sinai School of Medicine, New York, NY, USA; 2 Department of Community Medicine, Mount Sinai School of Medicine, New York, NY, USA; 3 Department of Medicine, Mount Sinai School of Medicine, New York, NY, USA; 4 A.I. DuPont Hospital for Children, Wilmington, Delaware, USA; 5 Leiden University Medical Center, Leiden, The Netherlands; 6 Columbia University, New York, NY, USA

## Abstract

**Aims:**

Patients with prior major cardiovascular or cerebrovascular events (MACE) are more likely to have future recurrent events independent of traditional cardiovascular disease risk factors. The purpose of this study was to determine if patients with traditional risk factors and prior MACE had increased cardiovascular magnetic resonance (CMR) plaque burden measures compared to patients with risk factors but no prior events.

**Methods and Results:**

Black blood carotid and thoracic aorta images were obtained from 195 patients using a rapid extended coverage turbo spin echo sequence. CMR measures of plaque burden were obtained by tracing lumen and outer vessel wall contours. Patients with prior MACE had significantly higher MR plaque burden (wall thickness, wall area and normalized wall index) in carotids and thoracic aorta compared to those without prior MACE (Wall thickness carotids: 1.03 ± 0.03 vs. 0.93± 0.03, p = 0.001; SD wall thickness carotids: 0.137 ± 0.0008 vs. 0.102 ± 0.0004, p < 0.001; wall thickness aorta: 1.63 ± 0.10 vs. 1.50 ± 0.04, p = 0.009; SD wall thickness aorta: 0.186 ± 0.035 vs. 0.139 ± 0.012, p = 0.009 respectively). Plaque burden (wall thickness) and plaque eccentricity (standard deviation of wall thickness) of carotid arteries were associated with prior MACE after adjustment for age, sex, and traditional risk factors. Area under ROC curve (AUC) for discriminating prior MACE improved by adding plaque eccentricity to models incorporating age, sex, and traditional CVD risk factors as model inputs (AUC = 0.79, p = 0.05).

**Conclusion:**

A greater plaque burden and plaque eccentricity is prevalent among patients with prior MACE.

## Background

Atherosclerosis is a major cause of morbidity and mortality world-wide with the most serious outcomes being myocardial infarction, stroke and death [1,2]. Atherosclerosis affects all vascular beds, including the coronary, carotid, aorta and peripheral arteries and is present years before a cardiovascular event[3]. Surrogate measures of atherosclerosis are increasingly being used as substitute endpoints for clinical trials, with serum biomarkers and imaging [4,5] being potential candidates for assessing underlying disease [6,7]. Surrogate markers for atherosclerosis if validated, might allow assessments of the effectiveness of cardio-protective interventions at earlier time points with significant savings in cost. It is also known that patients with prior major cardiovascular and cerebrovascular events (MACE) are at increased risk for recurrent events independent of other risk factors [8-10]. Atherosclerotic plaques have been imaged and certain plaque characteristics may be associated with an increased risk for cardiovascular events. For example, studies using MRI have been used to image vulnerable plaques that exhibit thin fibrous caps overlying large necrotic lipid cores [11,12]. High-resolution cardiovascular magnetic resonance (CMR) has also been shown to be capable of identifying plaque constituents, such as the necrotic core and intraplaque hemorrhage, in human carotid atherosclerosis [13].

Furthermore, although the underlying pathological processes might be similar in all vascular beds, there are other important factors such as endothelial shear stress which differ from one vascular bed to another [14,15]. Studies have evaluated the degree to which measures of subclinical atherosclerosis in multiple vascular beds correlate with each other and have reported the risk factors associated with atherosclerosis in each of these vascular beds [16]. Therefore, evaluating multiple vascular beds in an individual might provide additional information regarding their future risk for cardiovascular events.

The recent ENHANCE (Effect of Combination Ezetimibe and High-Dose Simvastatin vs. Simvastatin Alone on the Atherosclerotic Process in Patients with Heterozygous Familial Hypercholesterolemia) clinical trial recently compared the mean change in the intima-media thickness (IMT) measured at three sites in the carotid arteries between patients with heterozygous familial hypercholesterolemia treated with ezetimibe/simvastatin 10/80 mg versus patients treated with high-dose simvastatin 80 mg alone [17]. The results of this study may indicate that use of carotid intima media thickness (IMT) as a surrogate marker for rate of clinical events due to atherosclerosis might not be ideal [18,19]. These results also indicate that additional thrusts may be desirable towards pursuing other surrogate markers for clinical endpoints due to atherosclerotic disease and for creating end points for clinical trials.

Although not currently used on a routine clinical basis, black blood in-vivo non-invasive CMR [20,21] is an accurate, highly reproducible technique for the imaging of the arterial wall [22]. It is capable of measuring the total burden of atherosclerotic disease in multiple vascular distributions and has been suggested as a reliable outcome measure in clinical trials[22] and epidemiological studies[23]. CMR findings have been extensively validated against pathology in ex vivo studies of carotid, aortic, and coronary artery specimens obtained at autopsy and using experimental models of atherosclerosis[14]. CMR offers unique advantages for the assessment of the carotid arteries and the thoracic aorta for quantification of atherosclerosis including the ability to provide highly reproducible measures [24-27] of anatomy and atherosclerosis burden without ionizing radiation.

The purpose of this study was to determine if we could identify morphometric features of atherosclerosis in individuals with risk factors with and without prior MACE, which confer the historically known higher risk for recurrent events. We also sought to test whether including such CMR measures in a model for discriminating prior MACE can improve discerning power beyond a model consisting of demographics and traditional cardiovascular disease (CVD) risk factors. If differences were found in CMR plaque burden measures of the common carotid arteries and descending thoracic aorta in patients with and without prior MACE, this may provide preliminary information for the use of these measures in prospective studies. Results of this study might also help in evaluating CMR as a modality to further screen patients prior to inclusion in studies with clinical end points by reducing the number of subjects required for adequate power.

## Methods

This study was approved by the institutional review board of the Mount Sinai School of Medicine and written informed consent was obtained from all subjects. This study also conformed to the Health Insurance Portability and Accountability Act (HIPAA) guidelines.

### Patient population

One hundred and ninety five patients aged 50 years or older and with at least two risk factors for atherosclerosis were recruited serially from January 2003 – December 2006 into the imaging study. Patients were recruited from the offices of local physicians in the New York area and at Mount Sinai Hospital. The local physicians screened patients based on their traditional risk factors for atherosclerosis (age, gender, smoking status, hypertension, diabetes, lipid profile, and carotid intima-media thickness measures (IMT)). Any patient who had at least two risk factors for atherosclerosis (not including older age) was recruited into the imaging study. The following criteria were used to determine a positive risk factor: A mean IMT > 1.1 mm, or a focal structure that encroached into the arterial lumen by at least 0.6 mm represented a positive IMT. A patient was considered to be hypertensive if either systolic or diastolic blood pressure was greater than 140 and 100 mm HG respectively or the patient was on antihypertensive medication. A patient was considered to be hypercholesteromic if total cholesterol was > 200 mg/dl, and LDL was > 160 mg/dl or if the patient was on a statin. Fasting plasma glucose levels of more than 126 mg/dl on two or more tests on different days or HbA1c > 6.5 indicated diabetes.

### MR system

MR was performed on a 1.5T Siemens Sonata (Siemens Medical Solutions, Erlangen, Germany) whole body scanner running a Numaris 4.0 operating system. This scanner has a maximum gradient amplitude of 40 mT/m and slew rate of 200 mT/m/ms. The integrated body coil was used for transmission while a custom built 4-channel carotid array was used for signal reception of the carotid images. Aortic images were obtained using a 6 channel cardiac coil in conjunction with the spine array for signal reception.

### Carotid imaging protocol

Twelve non-overlapping cross sectional slices starting at and extending below the left carotid bifurcation were obtained using a the rapid extended coverage double inversion recovery turbo spin echo black blood (REX) pulse sequence developed previously [21]. Proton density images were obtained using the following parameters. Twelve slices were acquired simultaneously with a repetition time of 2130 ms and an echo time of 5.6 ms. A field of view of 12 × 12 cm was used in conjunction with a receiver bandwidth of 488 Hz/pixel, and a matrix size of 256^2^, a turbo factor of 15 and 2 signal averages. The slice thickness was 3 mm. Fat suppression was achieved using a chemical shift selective pulse. Acquisition time was approximately 3 minutes for each 12 slices imaged. Cardiac or peripheral gating was not used for carotid image acquisition [20]. Data from the left and right carotid arteries were combined and mean values used in the analyses.

### Thoracic Aorta imaging protocol

Sixteen transverse images just above the level of the diaphragm (scout images obtained at end expiration) were obtained using the REX sequence. The details of the REX sequence have been published previously[21]. All aortic images were obtained using cardiac gating. The imaging parameters were similar to those used for carotid imaging except as follows: 16 slices were acquired simultaneously with a field of view used of 20 cm^2 ^and slice thickness of 5 mm. Acquisition time was approximately 3 minutes for each 16 slices imaged. Respiratory gating was not used. The images were of acceptable quality without the use of respiratory gating and therefore it was deemed unnecessary. During the scan, if the image quality was deemed to be poor due to respiratory motion, the patient was asked to regulate his/her breathing and the scan was repeated. The use of multiple signal averages also reduced the effect of respiratory motion on the image.

### MR imaging analysis

After acquisition, MR images were transferred to a dedicated workstation for analysis. Each image was qualitatively assessed on a scale of 1–5, in four categories (overall image quality, flow suppression, artifacts, and vessel wall delineation) with 5 representing the best quality, by an expert observer (KBW, 5 years MR imaging experience) as described in earlier studies [20]. All images with an image quality of 1 or 2 in any of the four categories were excluded from analysis. On all images that were deemed to be of sufficient quality, the inner and outer vessel wall boundaries were manually traced for both the common carotids and thoracic descending aorta. Mean lumen diameter, vessel diameter, lumen area, wall area, total vessel area, mean wall thickness and standard deviation of wall thickness for each slice were calculated based on the contours drawn by the expert observer using a customized software program (Vessel Mass Software, Leiden University Medical Center, The Netherlands). The lumen diameter and outer vessel diameter were measured as follows: The centroid of the lumen and outer wall boundary were determined based on the contours drawn by the observer. Radial lines were drawn at 4° increments through the centroid towards the manually drawn contours. The length of the radial lines extending to the lumen boundary determines the lumen diameter, and the length of the radial line extending to the outer contours determines the outer vessel diameter. The average of the 45 radial measures of lumen diameter and outer vessel diameter are used for analysis.

**Figure 1:**
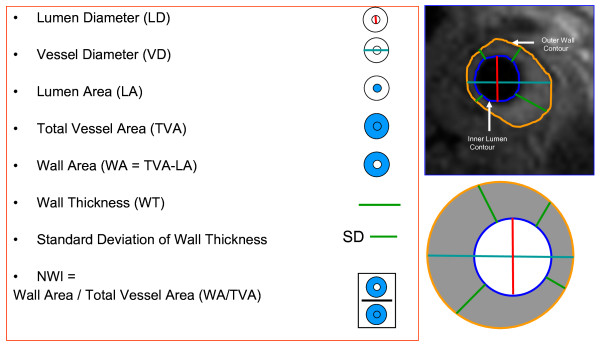
**MR imaging parameters measured for carotids and thoracic aorta**. The red line indicates the measure of the lumen diameter; the teal line represents the vessel diameter, the green line represents the wall thickness. The area enclosed by the blue contour represents the lumen area and the area enclosed by the orange contour represents the total vessel area. The difference between the total vessel area and the lumen area provides the wall area. The normalized wall index is determined by the ratio of the wall area to the total vessel area. The top right panel shows the contours on a sample carotid image.

**Figure 2:**
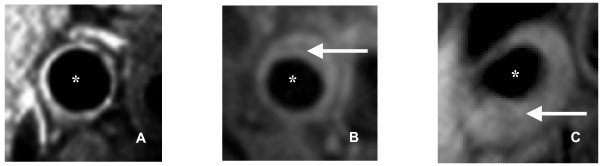
**Sample MR Images showing individuals with varying SD wall thickness of carotid arteries**. (Panel A shows an individual with high SD of wall thickness, Panel B shows medium SD of wall thickness and Panel C shows low SD of wall thickness).* indicates lumen. Arrow indicates plaque.

Wall thickness measurements were based on the Centerline method, a previously used technique [28]. Using this method, local wall thickness measurements were obtained at 100 evenly spaced positions along the circumference of the vessel wall. The standard deviation of the 100 measures of wall thickness provides the SD of the wall thickness measures (eccentricity of plaque) for that particular slice. The MR imaging parameters measured are shown in Figure 1. The normalized wall index (NWI) was used to account for differences in size of the arteries within each patient by normalizing to the outer vessel area. Several studies have previously used the NWI in an attempt to normalize wall areas for patient size [29,30]. The median number of slices available for each vascular bed for each individual analyzed was 5. Figure 2 shows sample MR images indicating plaques with varying standard deviation of wall thickness in carotid arteries. Wall volumes were not measured as only 2D imaging was performed and measures of average wall area and total volume reflect the same parameter (i.e. wall volume = average wall area * number of slices).

The individual performing the image contouring (KBW) was blinded to all patient history and demographic information including presence or absence of prior MACE. The order of patient presentation for contouring was randomized.

### Statistical Analysis

The cohort was stratified by the presence of prior MACE defined as stroke (including transient ischemic attacks) or coronary artery disease (including acute coronary syndrome, history of myocardial infarction, history of coronary artery bypass surgery, or history of percutaneous intervention). Demographic characteristics, traditional CVD risk factors, and CMR measures of the carotid arteries and thoracic descending aorta for the study population were summarized separately for those with and without prior MACE. Non-normally distributed variables were log transformed. Continuous variables were summarized using means, and geometric means for non-normally distributed variables, with differences across presence of prior MACE assessed using Student's t-tests. Dichotomous variables were summarized as proportions and compared using Chi-square tests.

For all analyses, MR measures of the carotid arteries and thoracic descending aorta were analyzed separately. The prevalence of prior MACE was correlated by tertile of each MR measure. Trends in prevalence across tertiles were calculated by modeling the median MR measure for each tertile as an independent continuous variable. The prevalence odds ratio for prior MACE associated with each MR measure, modeled as a continuous variable, was also computed. Initially, this association was determined after age and sex adjustment. Subsequent models included additional adjustment for traditional CVD risk factors including history of smoking, body mass index, hypertension and diabetes mellitus. Odds ratios are presented for a one standard deviation higher MR measure.

Next, the area under the receiver operating characteristic curves (AUC) for discriminating prior MACE was computed. An initial model assessed the discriminatory value of age and sex. A subsequent model included age and sex and traditional CVD risk factors with final models including age, sex, traditional CVD risk factors and each MR imaging measure, separately. The discriminatory ability of these models to detect those with prior MACE (i.e., the AUC) was compared following the method of DeLong, DeLong and Clarke-Pearson for correlated data[31] Statistical analyses were performed using Stata version 10.0 (Stata Incorporated, College Station, TX).

## Results

Characteristics of study participants by prior MACE are provided in Table 1. Participants with prior MACE were older, and had a history of diabetes mellitus.

**Table 1:** Characteristics of study participants by cardiovascular disease status.

	MACE		
	**Yes** **(n = 53)**	**No** **(n = 142)**	P-value

**Demographics**			

Age, years	65.3 (1.1)	60.1 (0.6)	**< 0.001**

Male, %	73.6	59.2	0.063

Ever smoking, %	54.7	57.0	0.771

Hypertension, %	54.7	43.0	0.056

On statin treatment %	82.0	67.2	0.081

History of diabetes mellitus%	41.5	24.2	**0.020**

Body mass index, kg/m^2^	26.9 (0.7)	26.9 (1.1)	0.993

**MRI parameter-Carotids**			

Lumen diameter^†^, mm	6.64 (6.43 – 6.84)	6.52 (6.39 – 6.65)	0.351

Vessel diameter^†^, mm	8.72 (8.47 – 8.98)	8.41 (8.26 – 8.57)	**0.042**

Wall thickness^†^, mm	1.03 (0.97 – 1.09)	0.93 (0.91 – 0.96)	**0.001**

SD wall thickness, mm	0.137 (0.008)	0.102 (0.004)	**< 0.001**

Lumen area^†^, mm^2^	0.348 (0.327 – 0.370)	0.335 (0.322 – 0.348)	0.332

Total vessel area^†^, mm^2^	0.60 (0.57 – 0.64)	0.56 (0.54 – 0.58)	**0.038**

Wall area^†^, mm^2^	0.25 (0.23 – 0.27)	0.22 (0.21 – 0.23)	**0.003**

Normalized wall index	0.416 (0.008)	0.396 (0.004)	**0.015**

**MRI parameter-Thoracic aorta**			

Lumen diameter, mm	22.1 (0.3)	22.0 (0.2)	0.833

Vessel diameter, mm	25.5 (0.4)	25.1 (0.2)	0.385

Wall thickness^†^, mm	1.63 (1.53 – 1.73)	1.50 (1.46 – 1.55)	**0.009**

SD wall thickness^†^, mm	0.186 (0.151 – 0.228)	0.139 (0.125 – 0.155)	**0.009**

Lumen area, mm^2^	3.90 (0.12)	3.87 (0.07)	0.801

Total vessel area, mm^2^	5.17 (0.16)	5.01 (0.09)	0.345

Wall area^†^, mm^2^	1.21 (1.12 – 1.32)	1.11 (1.07 – 1.15)	**0.027**

Normalized wall index	0.244 (0.006)	0.229 (0.003)	**0.016**

Numbers in table represent mean (standard deviation), ^†^geometric mean (95% confidence interval) or percentage

### Carotid imaging

Vessel diameter, wall thickness, plaque eccentricity (standard deviation of wall thickness), wall area, total vessel area, and normalized wall index were each significantly higher for individuals with prevalent prior MACE compared to those without (Table 1). Prior MACE was more common at each higher tertile of vessel diameter, wall thickness, plaque eccentricity, total vessel area, and wall area (Table 2).

**Table 2:** Prevalence and adjusted odds ratios of MACE associated with each tertile of magnetic resonance imaging parameters.

	Tertile of Magnetic Resonance Imaging Parameters			
	Tertile 1	Tertile 2	Tertile 3	P-trend

**Carotids**				

Lumen diameter, range in mm	< 6.20	6.20 – 6.83	> 6.83	

Prevalence of MACE	20.0	33.9	27.7	0.325


Vessel diameter, range in mm	< 8.15	8.15 – 8.86	> 8.86	

Prevalence of MACE	16.9	27.7	36.9	**0.011**


Wall thickness, range in mm	< 0.891	0.891 – 1.00	> 1.00	

Prevalence of MACE	18.5	23.1	40.0	**0.007**


Standard deviation wall thickness, range in mm	< 0.088	0.088 – 0.127	> 0.127	

Prevalence of MACE	12.9	29.0	43.6	**< 0.001**


Lumen area, range in mm^2^	< 0.302	0.302 – 0.369	> 0.369	

Prevalence of MACE	20.0	32.3	29.2	0.238


Total vessel area, range in mm^2^	< 0.522	0.522 – 0.619	> 0.619	

Prevalence of MACE	16.9	27.7	36.9	**0.011**


Wall area, range in mm^2^	< 0.204	0.204 – 0.244	> 0.244	

Prevalence of MACE	15.4	27.7	38.5	**0.004**


Normalized wall index, range	< 0.382	0.382 – 0.418	> 0.418	

Prevalence of MACE	24.6	20.0	36.9	0.117


**Thoracic aorta**				

Lumen diameter, range in mm	< 21.1	21.2 – 23.2	> 23.2	

Prevalence of MACE	26.2	26.2	29.2	0.693


Vessel diameter, range in mm	< 24.3	24.3 – 26.3	> 26.3	

Prevalence of MACE	24.6	27.7	29.2	0.555


Wall thickness, range in mm	< 1.43	1.43 – 1.63	> 1.63	

Prevalence of MACE	18.5	27.7	35.4	**0.032**


Standard deviation wall thickness, range in mm	< 0.123	0.123 – 0.199	> 0.199	

Prevalence of MACE	18.8	21.9	42.2	**0.004**


Lumen area, range in mm^2^	< 3.51	3.51 – 4.22	> 4.22	

Prevalence of MACE	26.2	26.2	29.2	0.693


Total vessel area, range in mm^2^	< 4.65	4.65 – 5.47	> 5.47	

Prevalence of MACE	24.6	27.7	29.2	0.555


Wall area, range in mm^2^	< 1.05	1.05 – 1.25	> 1.25	

Prevalence of MACE	21.5	26.2	33.9	0.112


Normalized wall index, range	< 0.214	0.214 – 0.241	> 0.241	

Prevalence of MACE	18.5	24.6	38.5	**0.011**

Abbreviations: mm – millimeter, MACE: prior major cardiovascular and cerebrovascular events

After age and sex adjustment, the prevalence odds ratios for prior MACE for each one standard deviation increase (0.19 mm for wall thickness, and 0.05 mm for plaque eccentricity) were 1.46 (95% CI: 1.03 – 2.06) and 1.66 (95% CI: 1.17 – 2.37), respectively (Table 3). After further adjustment for ever-smoking status, body mass index, hypertension and diabetes mellitus, the odds ratios for one standard deviation higher wall thickness (0.05 mm) became 1.80 (95% CI: 1.18–2.76).

**Table 3:** Odds ratios (95% confidence intervals) for MACE associated with one standard deviation higher magnetic resonance imaging parameters of carotid arteries, adjusted for age, sex, history of ever smoking, body mass index, hypertension and diabetes mellitus.

MRI parameter	Age and sex adjusted	Multivariate adjusted
**Carotids**		

Lumen diameter, 0.77 mm	1.05 (0.75 – 1.47)	1.03 (0.71 – 1.48)

Vessel diameter, 0.95 mm	1.21 (0.85 – 1.71)	1.18 (0.80 – 1.72)

Wall thickness, 0.19 mm	1.46 (1.03 – 2.06)*	1.47 (1.00 – 2.18)

Standard deviation wall thickness, 0.05 mm	1.66 (1.17 – 2.37)**	1.80 (1.18 – 2.76)**

Lumen area, 0.08 mm^2^	1.04 (0.75 – 1.44)	1.03 (0.72 – 1.46)

Total vessel area, 0.13 mm	1.20 (0.85 – 1.69)	1.18 (0.81 – 1.72)

Wall area, 0.06 mm^2^	1.36 (0.98 – 1.90)	1.36 (0.93 – 1.97)

Normalized wall index, 0.05	1.31 (0.94 – 1.82)	1.33 (0.92 – 1.93)

**Thoracic aorta**		

MRI parameter	Age and sex adjusted	Multivariate adjusted

Lumen diameter, 2.44 mm	0.86 (0.60 – 1.24)	0.87 (0.57 – 1.31)

Vessel diameter, 2.67 mm	0.94 (0.66 – 1.34)	0.97 (0.65 – 1.44)

Wall thickness, 0.35 mm	1.23 (0.87 – 1.73)	1.27 (0.89 – 1.83)

Standard deviation wall thickness, 0.11 mm	1.36 (0.99 – 1.87)	1.42 (1.00 – 2.03)

Lumen area, 0.85 mm^2^	0.87 (0.60 – 1.25)	0.88 (0.58 – 1.33)

Total vessel area, 1.07 mm	0.95 (0.66 – 1.36)	0.99 (0.66 – 1.47)

Wall area, 0.35 mm^2^	1.15 (0.82 – 1.62)	1.22 (0.85 – 1.75)

Normalized wall index, 0.04	1.34 (0.95 – 1.90)	1.41 (0.96 – 2.07)

Abbreviations: MRI – magnetic resonance imaging

* p < 0.05, ** p < 0.01.

**Figure 3:**
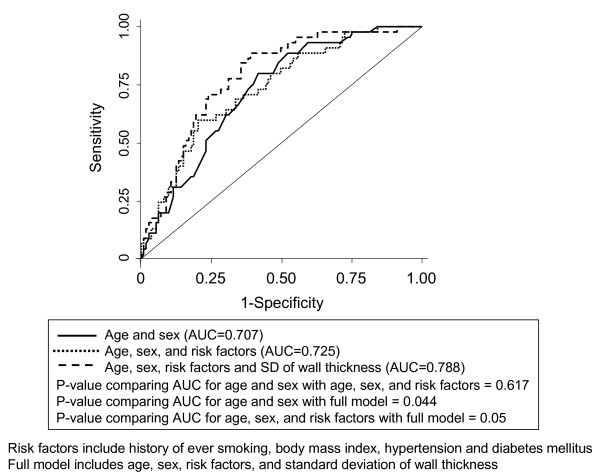
**Receiver operating characteristic curves for predicting cardiovascular disease using various models**.

The AUC for discriminating prior MACE using age and sex alone was 0.71, which increased to 0.73 by the additional inclusion of traditional CVD risk factors (Table 4). The only MR parameter that significantly improved the AUC was a measure of plaque eccentricity, i.e. the standard deviation of wall thickness; the AUC then became 0.79 (p-value = 0.05 compared to the age, sex, traditional risk factor model). Receiver operating characteristic curves for discriminating prior MACE for models comprised of age and sex, age, sex and traditional CVD risk factors, and these factors plus plaque eccentricity is also shown in Figure 3.

**Table 4:** Area under the receiver operating characteristic curves

**Model**	**Carotids**	**Thoracic aorta**
1) Age and sex*	0.71 (0.64 – 0.78)	0.71 (0.64 – 0.78)

2) Age, sex and risk factors†	0.73 (0.65 – 0.79)	0.73 (0.65 – 0.79)

3) Age, sex, risk factors^†^, and lumen diameter	0.73 (0.66 – 0.80)	0.73 (0.65 – 0.79)

4) Age, sex, risk factors^†^, and vessel diameter	0.73 (0.66 – 0.80)	0.72 (0.65 – 0.79)

5) Age, sex, risk factors^†^, and wall thickness	0.75 (0.68 – 0.81)	0.74 (0.66 – 0.80)

6) Age, sex, risk factors^†^, and standard deviation wall thickness	**0.79 (0.71 – 0.85)**	0.74 (0.66 – 0.80)

7) Age, sex, risk factors^†^, and lumen area	0.73 (0.65 – 0.79)	0.72 (0.65 – 0.79)

8) Age, sex, risk factors^†^, and total vessel area	0.73 (0.66 – 0.80)	0.72 (0.65 – 0.79)

9) Age, sex, risk factors^†^, and wall area	0.74 (0.66 – 0.80)	0.73 (0.66 – 0.80)

10) Age, sex, risk factors^†^, and normalized wall index	0.74 (0.66 – 0.80)	0.74 (0.67 – 0.81)

Numbers in the table represent AUC and 95% confidence interval, Bold represents highest AUC value (See Figure 3 for p-value)

AUC – Area under the ROC curve

† Risk factors include history of ever smoking, body mass index, hypertension and diabetes mellitus.

### Thoracic aorta imaging

Wall thickness, plaque eccentricity, wall area, and normalized wall index of the thoracic aorta were each significantly higher among individuals with prior MACE (Table 1; bottom panel). Prior MACE was also more common at each progressively higher tertile of wall thickness, plaque eccentricity, and normalized wall index (Table 2; bottom panel).

Modeled as a continuous variable, plaque eccentricity and normalized wall index showed the highest, albeit non-significant, increase in the prevalence odds ratios of prior MACE after multivariate adjustment (Table 3; bottom panel). The bottom panel of Table 4 shows the AUC for discriminating prior MACE using models incorporating age, sex, traditional CVD risk factors and MR parameters. Compared to results of a model including age, sex, and traditional CVD risk factors, incorporating MR imaging data did not significantly improve the ability to discriminate prior MACE.

## Discussion

The results of the current study of non invasive imaging of two vascular beds (carotid arteries and the thoracic descending aorta), obtained during a single imaging examination, indicate that MR imaging parameters of carotid arteries provide additional information with respect to the differences in the nature of the atherosclerosis in subjects with traditional risk factors with and without prior MACE. Most vascular MR studies typically focus on only one arterial bed. The current study is unique in the respect that both the carotids and thoracic descending aorta were studied in the same patients during a single examination. In agreement with previous studies [32-34], individuals in our study with prior MACE were older, and more likely to have diabetes[35] than those without prior MACE.

In the current study, patients with prior MACE had significantly higher measures of plaque burden such as wall thickness, wall area and plaque eccentricity (standard deviation of wall thickness) but had similar lumen areas compared to individuals without prior MACE. This indicates potential positive "Glagov" remodeling [36] in both the carotid arteries and the thoracic aorta among individuals with prior MACE. Black blood MR imaging of atherosclerosis provides valuable information beyond the luminogram provided by contrast angiography. Also we showed that the standard deviation of the wall thickness of atherosclerotic plaques in the carotid arteries, a measure of the eccentricity of the lesion, is independently associated with increased odds of having prior MACE. The role of carotid plaque eccentricity as a measure that predicts previous MACE warrants further exploration with regard to determining a potential mechanistic link.

Standard deviation of the wall thickness was not significantly associated with prior MACE in the case of the thoracic aorta indicating that eccentric plaques in carotid arteries may portend a greater risk than eccentric plaques in the thoracic aorta.

Our study indicates that carotid MR imaging information improves discrimination of prior MACE when incorporated into a model that includes age, sex and traditional risk factors. Although not prospectively evaluated in our study, the measures of atherosclerosis which show the strongest associations with prior MACE (wall thickness and plaque eccentricity) over and above traditional risk factors may help explain some of the excess risk of future events that these subjects are known to suffer from in clinical observations. It appears from our study that carotid MR imaging might provide more useful information than thoracic aorta imaging for defining risk. This result correlates with previous investigations that showed that abdominal aortic imaging was more useful than thoracic aorta imaging for atherosclerotic disease [37-39]. Previous studies have also examined the effect of age and gender on CMR measures of plaque burden and have also assessed the inter and intra observer reproducibility of the imaging techniques proposed here [40]. This study showed that the CMR measurements of atherosclerotic plaque burden were reproducible and that the CMR values for the mean wall areas correlated strongly between carotid arteries and aorta, suggesting a systemic distribution of disease. Similar to other clinical variables for cardiovascular diseases, values for CMR parameters were found to be higher in men than in women [40].

### Limitations

One of the major limitations of this study is the cross-sectional nature of the data obtained. Because this data was not analyzed prospectively, any conclusions that can be drawn from this study can only be causal associations[41]. Another limitation of the study is the fact that all patients were followed up only by their respective primary care physicians and not as part of a research clinical management protocol. Other limitations include non-random selection of subjects, incomplete availability of demographic information (i.e., race and ethnicity) and lipid profiles. For the AUC analysis, some factors such as ethnicity, lipid profiles, were not used for developing models as this information was unavailable from all patients included in this study. However, lipid profiles in available patients were not significantly different between those with and without prior MACE. Only risk factor data obtained 3 months prior to or after the imaging scan was used for the analysis. As no patients were scheduled for a carotid endarterectomy, histological examinations of plaques could not be performed. Another potential limitation was that plaques were only classified based on burden measured by proton density weighted imaging and not their composition using multi contrast CMR and the fact that only carotids and thoracic aorta images were analyzed. Another limitation of the study could include the fact that only images of sufficiently high quality were included for analysis and this selection of images could introduce a selection bias into our dataset. Also, to reduce partial voluming effects and artifacts caused in MR measures due to poor flow suppression (that may artificially increase wall thickness measurements); only images from the common carotid were used for analysis thereby eliminating the carotid bifurcation, the region most susceptible to such effects. The carotid bifurcation however is also the location where atherosclerotic disease is more prevalent and poor flow suppression may be related to the cardiovascular health of the arteries being observed as well. All images were traced manually and could be subject to observer bias as well. An automated tracing program could have produced more robust and reproducible results in terms of vessel wall traces. However, several studies have shown that MR imaging is reproducible for the quantification of vessel and plaque areas and can be used confidently in studies, which require measuring plaque size[24,25,42]. Imaging data from right and left common carotid arteries were combined for analysis. Although flow mechanics for each artery might be different, an analysis of the left and right carotids produced markedly similar results.

## Conclusion

A greater plaque burden and plaque eccentricity in the carotid arteries is prevalent among patients with risk factors and prior MACE compared to those with risk factors only. Longitudinal studies are needed to further assess the value of these and other MR measures of atherosclerosis as prospective markers of future incident and recurrent events. Clinical trials may use MR imaging in addition to traditional risk factors to enrich their samples with subjects at higher risk for cardiac events.

## Competing interests

The authors declare that they have no competing interests.

## Authors' contributions

VM, PM, SSG, VF, MW and ZAF conceived and designed the study. KBW, HT traced the imaging contours. SHA, HEA compiled and managed the patient database and were involved in data assembly. MF, JEP, SB recruited patients for the study and followed up with their clinical care. JVDG, JHCR developed the software for the image analysis and provided its implementation. PM and MW performed the statistical analysis of the data. VM, SHA, SG were involved in image analysis, collection and interpretation of data. VM, PM and ZAF drafted the article. VM, PM, SSG, VF, MW and ZAF were responsible for critical revision of the article for important intellectual content. All authors read and approved the final manuscript. VM and ZAF are the guarantors of the integrity of the study.
